# Methodological advancements in costing methods for (public) health economic evaluations: results from the European PECUNIA project

**DOI:** 10.1093/eurpub/ckac129.238

**Published:** 2022-10-25

**Authors:** J Simon

**Affiliations:** 1 Department of Health Economics, Center for Public Health, Medical University, Vienna, Austria; 2 Department of Psychiatry, University of Oxford, Oxford, UK

## Abstract

(Public) health economic evaluations face significant problems regarding the standardization and comparability of their methods. In addition, at least a quarter of the total direct cost impact of healthcare interventions affects other economic sectors. International methods and tools are lacking for the rigorous and comparable assessment of the costs and outcomes of (public) health care from a societal perspective. The H2020 PECUNIA project (grant No 779292) brought together ten partners from six countries (AT/DE/ES/HU/NL/UK) between 2018 and 2021 aiming to improve the comparability and feasibility of multi-sectoral, multi-national health economic evaluations in Europe. A multi-step, mixed methods approach was used following a new harmonized costing concept to develop new methods and tools for the standardised identification, definition, measurement and valuation of costs in multiple sectors (health care, social care, (criminal) justice, education, employment and productivity, and patient, family and informal care), and for the broader, harmonised, supra-national assessment of outcomes using selected mental disorders as illustrative examples. This presentation will summarise the relevant advances in costing methods, give an overview of the developed tools that are now publicly available (www.pecunia-project.eu/tools), and discuss the lessons learned regarding how far it is possible to harmonize costing evidence with standardised tools in Europe, and what the necessary future research directions may be.

